# Whole examination AI estimation of fetal biometrics from 20-week ultrasound scans

**DOI:** 10.1038/s41746-024-01406-z

**Published:** 2025-01-11

**Authors:** Lorenzo Venturini, Samuel Budd, Alfonso Farruggia, Robert Wright, Jacqueline Matthew, Thomas G. Day, Bernhard Kainz, Reza Razavi, Jo V. Hajnal

**Affiliations:** 1 School of Biomedical Engineering and Imaging Sciences, Faculty of Life Sciences and Medicine, King’s College London, London, UK; 2 Guy’s and St Thomas’ NHS Foundation Trust, London, UK; 3 Department of Computing, Faculty of Engineering, Imperial College, London, UK; 4 Department of Artificial Intelligence in Biomedical Engineering, FAU Erlangen-Nürnberg, London, Germany

**Keywords:** Ultrasonography, Intrauterine growth

## Abstract

The current approach to fetal anomaly screening is based on biometric measurements derived from individually selected ultrasound images. In this paper, we introduce a paradigm shift that attains human-level performance in biometric measurement by aggregating automatically extracted biometrics from every frame across an entire scan, with no need for operator intervention. We use a neural network to classify each frame of an ultrasound video recording. We then measure fetal biometrics in every frame where appropriate anatomy is visible. We use a Bayesian method to estimate the true value of each biometric from a large number of measurements and probabilistically reject outliers. We performed a retrospective experiment on 1457 recordings (comprising 48 million frames) of 20-week ultrasound scans, estimated fetal biometrics in those scans and compared our estimates to real-time manual measurements. Our method achieves human-level performance in estimating fetal biometrics and estimates well-calibrated credible intervals for the true biometric value.

## Introduction

Ultrasound (US) imaging is routinely used in many countries during pregnancy to screen for fetal abnormalities, often at ~18–22 weeks^1^. Procedures typically involve imaging of standard planes of fetal anatomy and measurement of several fetal biometrics, such as head circumference (HC) and femur length (FL). In the UK, requirements for screening examinations are published in the Fetal Anomaly Screening Programme (FASP) standard^2^.

Fetal biometrics are conventionally measured in single images: the operator pauses the US image stream on a specified view and then manipulates calipers to measure the anatomy. Some guidelines recommend that this whole procedure be repeated up to three times to ensure reliability^3^. Traditionally, these measurements have been done manually. Manual measurement of fetal biometrics displays significant expected-value bias^4^. Selection bias may also influence the planes used for measurement: different sonographers may systematically select different views of a given biometric, leading to repeatable differences in their measurements^5^. Furthermore, these manual processes display significant inter-observer variability, ranging from 4.9% to 11.1% across different common biometrics^5^. Early detection of several common fetal abnormalities remains low (often under 50% at second-trimester scans) and exhibits large geographic variation^6^, suggesting significant differences in workforce skills.

Recent research has proposed methods for automating the detection of standard anatomical planes during live scanning^7–10^ and biometric measurements^11^. US machine manufacturers are increasingly integrating these tools into their equipment^12^. However, fetal biometry generally still follows existing workflows that rely on operator selection of individual images. This does not take full advantage of the real-time nature of US, where a video stream of tens of frames per second is acquired. A fully automatic system could perform plane classification on every frame of the US stream and biometric measurement using all available data.

Full automation would reduce human selection bias from plane selection and expected-value bias during measurement, potentially improving veracity, reproducibility and reducing operator dependence. Automating some key operator tasks could also reduce total scan time as well as cognitive load, allowing the sonographer to focus more on the patient and on identifying signs of anomalies.

Evaluating every frame in a fully automatic system generates a very large number of measurements. A key challenge in making such a system useful for clinical practice, therefore, is to generate a single estimate from the vast resulting amount of information. There has been some work in developing methods to calculate fetal biometry from a video feed. Płotka et al.^13^ propose a system to select the best standard planes to extract biometry, and Lee et al.^14^ average convolutional neural network (CNN) outputs across a scan to estimate gestational age. Matthew et al.^15^ have proposed a system to automatically classify every frame in a US scan and extract fetal biometrics, but an operator still needs to select appropriate frames to report biometrics. In other ultrasound applications, Blanco et al.^16^ design a network architecture that takes multiple frames as input and returns a single output measurement, but this only extends to a limited window. These methods have varying performances relative to single-frame approaches, but they all adopt the principle of obtaining biometrics from a larger collection of images.

No work has, to our knowledge, sought to obtain an expected value of the biometry and a credible interval by combining all frame measurements while acknowledging potential outliers. This would represent a substantial departure from current clinical practice, which is fully focused on a selection and measurement of single images.

We propose a real-time system that can identify standard planes and estimate fetal biometrics achieving and reporting progressively reducing uncertainties. The system is designed to seamlessly link into clinical practice during 20-week US screening scanning. We build upon Sononet^7^ for standard plane detection, and pair it with automatic biometric estimation per frame, aggregating these to generate a progressive global estimate using a Bayesian framework. When used during live scanning, this results in progressively more reliable central estimates and credible intervals for each biometric. The proposed method has the potential to improve clinical practice by providing robust measurements free from expectation bias while reducing cognitive load on the sonographer.

## Results

### Experimental setup

We developed a set of neural networks to measure fetal biometrics at the 20-week ultrasound scan in real-time. In the UK FASP standard, 13 standard planes must be visualised and saved and at least 5 biometrics must be measured: the head circumference (HC), biparietal diameter (BPD), abdominal circumference (AC), femur length (FL) and transcerebellar diameter (TCD).

We used a large dataset of recordings of routine 20-week ultrasound scans (the iFIND1 dataset, described in the Methods section) to train CNNs to measure each of the required fetal biometrics. We also read the scale bar on each image automatically to extract a metric measurement of each biometric. These networks were trained and tested in individual ultrasound images.

We then integrated these CNNs into a full pipeline to estimate each fetal biometric over the course of an entire ultrasound scan. First, we use the Sononet standard-plane classification network^7^ to classify each frame in an ultrasound video stream: most video frames are not standard planes and therefore require no further processing. Frames identified as a standard plane that contains biometrics are measured using our biometric CNNs. This leads to a large number of measurements for each biometric: we propose a Bayesian method to obtain the best central estimate from all visible measurements over the course of an examination. The processing pipeline we used is described in more detail in our ‘Methods’ section.

### Experiments performed

The variability in biometric measurements between two sonographers arises from two components: image selection for measurement and caliper placement within each image. Previous work has shown that 50–80% of the variability between humans can be explained by caliper placement^5^, leaving 20–50% of variability accounted for by image selection. The proposed whole-scan method eliminates this second source of noise by processing every frame in a scan with no manual intervention and seeks to reduce the effect of random error in caliper placement by aggregating measurements from many individual frames.

We performed three experiments to quantify the reliability of our biometric estimates:

1. Using our biometric CNNs to independently measure the same frames that sonographers labelled. This tests the biometric measurement performance of our CNNs and compares it to published estimates of inter-rater differences in caliper placement on a single image. Note this is still a restricted problem, requiring human intervention to perform frame selection. For these single-image experiments, biometrics were estimated automatically in the same frames that sonographers chose to make their manual annotations. We trained one CNN per standard plane (from subjects in the training set) and tested it using the subjects in the test set using the unannotated copies previously extracted for all frames in the iFIND test set: 1516 in the brain-TV standard plane, 1360 for brain-CB, 1205 for abdominal, and 1124 for femur.
2. Aggregating biometric measurements across a whole recorded scan to obtain global estimates for the scan and comparing to sonographer manual measurements. This experiment considers a more unconstrained problem with no human interaction. It does not control for individual frame selection, so our results can be compared to inter-rater variability in biometric measurement from different scans. All 1457 recordings in the test set were used. The final estimate of each biometric, along with credible intervals, was reported.
3. Conducting a test-retest experiment on the paired scan data described in the ‘Methods’ section. We ran the full pipeline on paired scans of the same subject acquired on the same day using a different scanner from the training dataset. We then compared biometrics from each scan and measured test–retest variability of our algorithm. The scans here were performed on a US machine by a different manufacturer from the training data, which allowed us to examine domain shift.

We also analysed data from our experiments to validate our modelling of the measurement distribution. Our approach models the distribution of measurements of each biometric with a mixture of a uniform distribution and a Gaussian distribution

1 $$D={P}_{t}{\mathcal{N}}\left(\mu ,{\sigma }^{2}\right)+(1-{P}_{t})U(a,b).$$

This has three parameters *P*_*t*_, *μ* and *σ* which are tuned iteratively to incoming data. More details and justification of this model can be found in the ‘Methods’ section. This model assumes that incoming measurements follow a Gaussian distribution (if correctly classified) or a uniform distribution (if noise), which can be tested. We, therefore, examined the output biometric measurements to check the appropriateness of this model.

### Single-frame biometric estimation

A valid biometric estimate (following the constraints outlined in our ‘Methods’ section) was obtained in approximately 90% of cases across all biometrics. Table 1 shows performance on the unlabelled copies of the frames labelled by sonographers in the test dataset. None of the measured biases were statistically significant except for AC, which exhibited a small positive bias in its measurement (*p* < 0.01).

**Table 1:** Comparison of the biometric measurements obtained on the test set of the iFIND dataset

Structure	Bias (mm)	MSD (mm)	Human MSD
HC	+0.11 mm (+0.07%)	4.34 mm (2.68%)	2.70 mm (1.8%)
BPD	+0.06 mm (+0.17%)	1.28 mm (3.20%)	N/A
AC	+1.51 mm (+0.98%)	5.03 mm (3.26%)	4.03 mm (2.8%)
FL	0.00 mm (0.00%)	1.53 mm (4.67%)	1.02 mm (2.9%)
TCD	−0.16 mm (−0.75%)	2.1 mm (10.1%)	N/A

‘Bias’ refers to the average difference in measurement between our method and human sonographers. ‘MSD’ is the mean-squared difference between pairs of measurements. The ‘Human MSD’ column is populated with values of inter-observer variability reported by Sarris et al.^5^.

Using the sonographer measures as the reference point, the automated single frame performance showed a bias of less than 1% and a mean square difference (MSD) of less than 5%, except for TCD, which showed an MSD of just over 10%. Where inter-observer data is available for manual measurements, the automated approach could be seen to have an average of 41% lower agreement with the human rater.

### Whole-scan processing

**Fig. 1:**
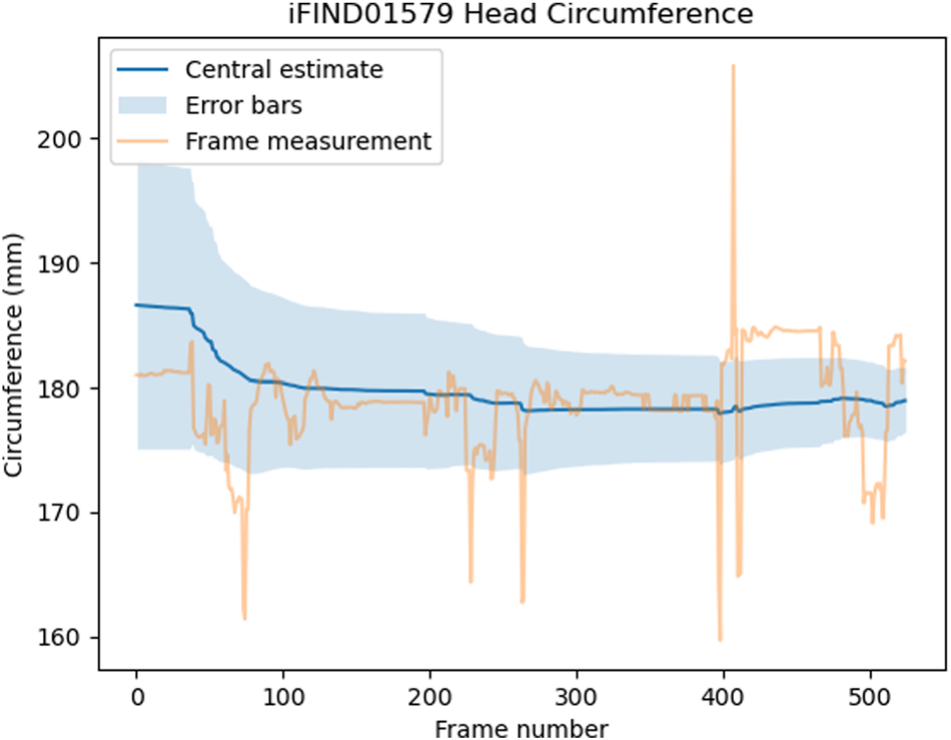
Evolution of the HC biometric estimate over time in an individual subject. Each individual frame measurement (yellow) is integrated into the method for calculating a single overall estimate (blue). Each measurement makes a small contribution. Measurements which are very far from the credible intervals are considered likely to be misclassifications and given a lower weight.

**Fig. 2:**
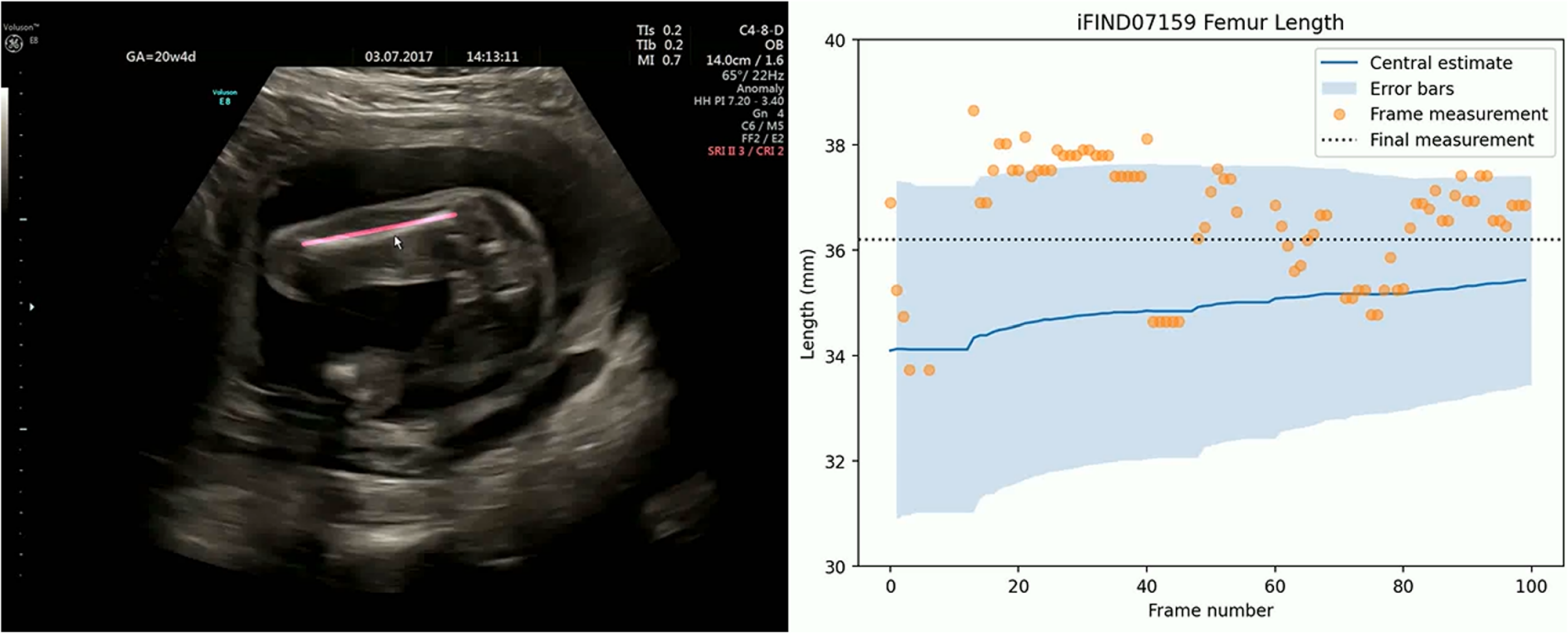
A video showing real-time measurement of biometrics and updating of the biometric estimate. In three seconds of real-time scanning (at 30 frames per second), our system acquired 83 FL measurements and updated its FL estimate from 34 mm (the prior, based on the average FL for this gestational age) to 36 mm. Seven frames did not have a visible FL measurement and did not contribute to the estimate. The full video clip can be found at http://www.ifindproject.com/wp-content/uploads/sites/79/2023/12/realtime.gif. In the PDF version of this article, please click anywhere on the figure or caption to play the video in a separate window.

Figure 1 shows how a biometric estimate (in this case, HC) changes over time during one exemplar scan, overlaid with measurements from individual frames. Initially, the prior dominates, estimating the 50th percentile for the age with wide error bars. As the number of frames contributing measurement values increases, the overall estimate becomes more stable and resistant to outliers. Although individual frame measurements show significant noise, including extreme values (which are probabilistically discounted by our system), the credible interval progressively shrinks with increasing data. Figure 2 shows this happening in real time over 3 s of scanning.

**Fig. 3:**
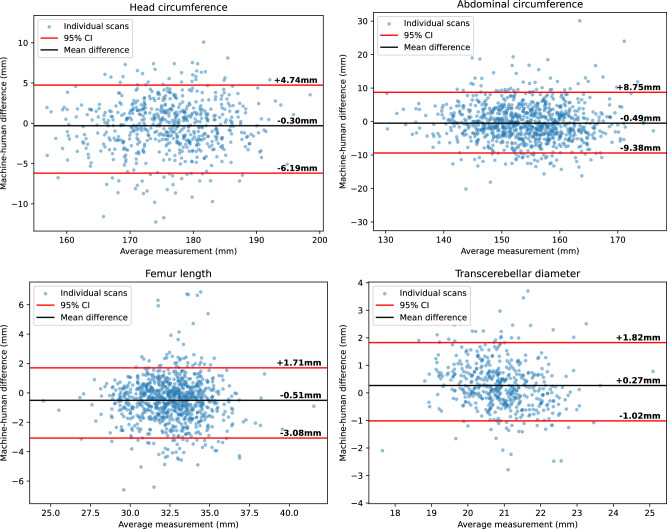
Bland–Altman plots showing the differences between the sonographer’s measurements and our own across all complete recordings in the test dataset. Our estimates, based on measurements acquired across an entire scan show good agreement with those of human sonographers across the dataset. The inter-rater variability is similar to that between humans.

Table 2 shows the performance of our biometric estimation across a whole video, and Fig. 3 shows Bland-Altman plots for human and machine measurement on the same subjects for three biometrics, with 95th percentile intervals overlaid for three biometrics. The difference between human measurements (taken from sonographers’ reports after each scan) and machine estimates can be compared to inter-rater disagreement measured by Sarris et al.^5^. Our 95th percentile intervals are overall very similar to human inter-rater variability for HC, AC and FL. 95.1% of machine–human measurement differences (across these three biometrics) lie within the 95% range of human differences.

**Table 2:** Comparison of human measurements to machine estimates taken across a whole scan

Biometric	Bias	MSD	MAD (mm)	Std. err (mm)	Within human 95%
HC	−0.30 mm (−0.18%)	3.23 mm (1.99%)	2.50 mm (1.42%)	3.39 mm	94.7%
BPD	+0.90 mm (+1.89%)	1.86 mm (4.65%)	1.11 mm (2.31%)	0.95 mm	N/A
AC	−0.49 mm (−0.31%)	5.55 mm (3.60%)	4.23 mm (2.74%)	3.26 mm	96.6%
FL	−0.51 mm (−1.59%)	1.63 mm (4.99%)	1.19 mm (3.64%)	1.12 mm	93.9%
TCD	+0.27 mm (+1.32%)	0.91 mm (4.37%)	0.68 mm (3.28%)	0.47 mm	N/A

‘MAD’ refers to the mean absolute difference between human and machine measurements. The ‘within human 95%’ column shows the percentage of biometrics in the test set where the human–machine difference is within 95% of human-human inter-observer differences (taken from Sarris et al.^5^).

### Paired scan data

We also conducted a test-retest experiment to measure reliability across repeated scans of the same subject.

Table 3 shows the mean-square difference (MSD) and the standard deviation of the difference in the final biometric estimates from the test-retest experiment using data from 20 subjects scanned twice on the same day by different sonographers, described in more detail in our ‘Methods’ section below. We measured the per cent standard deviation in this table to ensure comparability with the measurements reported by Sarris et al.^5^, who reported human standard deviation. They examined a slightly different gestational age range, so we report a per cent standard deviation to normalise for that. Not all pairs of scans had sufficient frames visible of each biometric in both scans: the number of pairs available for analysis is shown in the table.

**Table 3:** Test–retest reliability of our model outputs from paired scans of the same subject

Biometric	MSD	Standard deviation	Human s.d. (full scan)	Number of pairs
HC	2.60 mm (1.25%)	1.23%	2.75%	15
BPD	0.79 mm (1.36%)	1.17%	N/A	15
AC	4.61 mm (2.51%)	2.50%	5.05%	17
FL	0.97 mm (2.44%)	2.44%	5.56%	10
TCD	0.57 mm (2.33%)	1.62%	N/A	8

The ‘Human s.d.’ column is populated with results by Sarris et al.^5^.

### Measurement distributions

The distribution of the resulting measurements was also examined to determine whether the model used to make biometric estimates was appropriate. The values were demeaned and then aggregated across all scans. We examined the distribution of all measurements across the test dataset. The values were demeaned by subtracting the final estimate for each subject from all measurements performed on that subject and then aggregated across all scans.

**Fig. 4:**
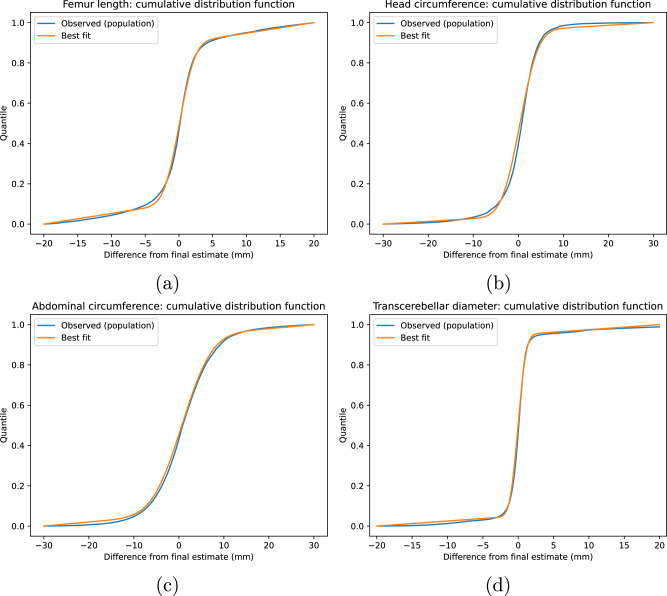
Cumulative distribution functions of all measurements for each examined biometric overlaid with the equivalent distribution for a fitted model using a mixed Gaussian + uniform distribution. We modelled the distribution of measurements of each biometric (**a** FL, **b** HC, **c** AC, **d** TCD) as consisting of a superposition of a Gaussian distribution (consisting of correct measurements) and a uniform distribution (consisting of noise and misclassifications). This is in good agreement with the observed distributions.

Figure 4b shows the cumulative distribution function (cdf) of all measurements obtained that way across all biometrics, as well as the best-fit theoretical model distribution for this data. The global best-fit parameters for the femur length were *P*_*t*_ = 0.79, *μ* = 0 and *σ* = 1.8 mm.

## Discussion

Our experiments show that the proposed workflow can improve the accuracy and repeatability of biometric estimation. In single frames, our models generally produce more variable biometric estimates than humans. However, when estimating biometrics across an entire examination, our estimates are in line with those made by sonographers (machine–human MSD ~ human–human MSD). Paired scan data also shows that our results are highly repeatable, with little difference across scans of the same subject. This latter test is a direct analogue of inter-rater testing by human observers, and results were in closer agreement than for manual measurement.

In individual frames, our method is generally in good agreement with sonographer measurements, as shown in Table 1. Sarris et al.^5^ report inter-observer differences when two sonographers manually place calipers on the same US frame for three biometrics. When referenced to the corresponding human measurements, the machine measurements in this experiment across all biometrics show more divergence than humans do in a single frame. This appears to be a random error: there is little observed bias, except for a small statistically significant difference in AC measurements.

**Fig. 5:**
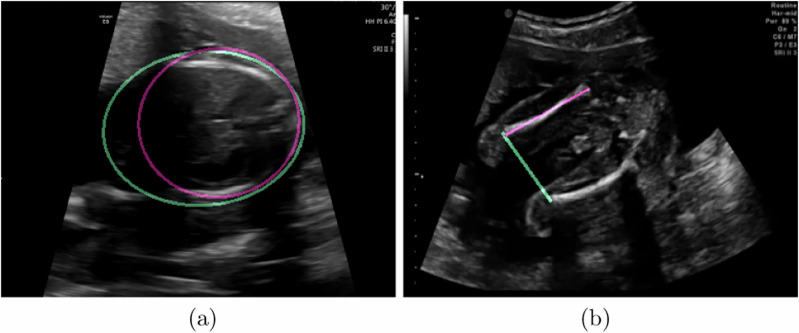
Examples of failure cases in biometric estimation. This figure shows two failure cases of biometric estimation in **a** the Head Circumference elliptical biometric and **b** the Femur Length linear biometric. The pink annotation shows the ground truth measurement made by a sonographer, while the green annotation is the automatic one made by our CNNs.

Part of this difference can be explained by the failure modes of our networks. Figure 5 shows some frames for which the models give an inaccurate biometric estimate. This typically occurs when structures of interest are poorly visualised in the US image or in some edge cases such as when both femurs are visible in a frame. These failure modes are uncommon (divergences of over 20% as shown in Fig. 5 are seen in only 1.5% of segmentations) but they are not mistakes that a human is likely to make.

There is significant variation in human-machine differences across biometrics. TCD shows a very large MSD of over 10%, while other biometrics are significantly more closely aligned to the human measurement. Human inter-observer variability in TCD measurements has been reported as being between 3 and 5%^17^, so the machine method appears to show significantly greater variability. In Supplementary Material C, we have also evaluated our results on the publicly available HC18 dataset^18^: we find good agreement, providing some external validity to our results.

We also evaluated the performance of our models across the length of an entire scan. The clinical standard for biometric measurement in fetal US relies on operator selection and annotation of a small number of individual frames. We take a different approach: we analyse every frame in which the biometry is visible and use all available measurements to generate a global estimate of the biometric. This avoids most operator dependence associated with the selection of standard planes, which can be a substantial contributor to inter-rater variability. Furthermore, it reduces the impact of random error from caliper placement in any one plane, which is also a significant source of variability.

The overall estimates of biometrics across a scan show better agreement with manual measurements than those from a single image. For instance, the TCD estimates across a scan show an average MSD with a manual measurement of 4.37%, compared to 10.1% for individual frames. This is despite the fact that the frames selected by the sonographers were removed: aggregating measurements from a diverse and unselected range of views of the anatomy results in a similar level of agreement with the sonographer. This value is also comparable with human inter-observer variability estimates found in the literature^17^.

There are also a few extreme outliers, demonstrating that our system achieves human-level performance. This is despite the fact that the performance of our CNNs on individual frames is lower than that achieved by humans: our whole-scan estimation method can compensate for the errors introduced by our single-frame estimation CNNs. We expect that our whole-scan algorithm could, therefore, achieve superhuman performance in biometric estimation if the single-frame estimator can be improved to the human level. The test-retest experiment we performed provides support for this, showing the variability in biometric estimates across scans is much lower than that between humans.

Furthermore, our method allows us to estimate credible intervals in which a given biometric can be expected to lie. This is based on the distribution of measurements and the number of observations, as described in our Methods section. On average, this is smaller than the MSD by a factor of 1.4. However, the manual measurement itself displays variability: assuming Gaussianity, MSD should be larger than the standard error by a factor of $\sqrt{2}\approx 1.4$. Therefore, the calculated standard error is in line with the observed variability in our estimates. Our credible intervals, therefore, provide a quantitative measure of the uncertainty in biometric estimation.

The system discussed in this paper treats single-frame measurements in a US scan as following a Gaussian distribution (when they are not due to misclassifications). The distributions of measurements examined in the ‘Results’ section show that this is globally an appropriate assumption, with measurements following the expected distribution. The distribution of measurements within each scan may be variable, depending on the operator’s choice of scanning planes, but the Bayesian estimation method ensures that an optimal estimate is always achieved given the available data.

Another advantage of this system, when applied in real-time, is that it requires no sonographer interaction to obtain biometrics. Much of a prenatal US scan is spent acquiring biometrics, and removing the need for this measurement can reduce scan time by up to a third^15^. A prospective trial of this system would be needed to estimate and quantify a time-saving.

We also performed experiments on paired data from the same subject. Despite a small number of pairs, across all biometrics our models are more repeatable and consistent than humans. The standard deviation of the difference between measurements of the same subject was approximately half of that between human sonographers. Much of the remaining difference can likely be explained by the different views of the relevant biometrics acquired in each scan determined by sonographer technique and fetal lie. This experiment was conducted in a sample scanned with a different US scanner and at a higher gestational age range than the training dataset, yet the model outputs remained robust to this domain shift. We have also conducted experiments using a public dataset in Supplementary Material C.

The generalisability of our method beyond the machines examined in this paper cannot be proven. While our methods may be robust to the domain shift introduced by a different machine discussed in this paper, we cannot extrapolate this to all models of ultrasound machines currently on the market. It is possible that other machines, by applying different types of data acquisition and preprocessing, may present sufficiently different data to degrade our methods’ accuracy. However, the approach we describe to generate a single overall estimate from a noisy set of measurements is independent of the method used to generate those estimates: this contribution presented in this paper should not be susceptible to domain shift.

If used in clinical practice, the method described in this paper could lead to a significant change in a sonographer’s workflow.

The method in this paper does not strictly require a minimum number of measured frames to generate an estimate: any number of measurements can return a central estimate, as well as a credible interval. If the measurements returned are very few or very noisy, for instance, due to an unfavourable fetal lie or poor technique by an acquiring sonographer, the credible interval returned for biometrics may be very wide and not clinically useful. To remedy this, a clinical application of this system may not show a real-time biometric estimate before the credible intervals have shrunk below a certain width. It may also require enough data to make the prior estimate insignificant, to ensure convergence.

While the results from this study suggest that our approach has accuracy similar to humans, caution should be taken in abnormal cases. Our training set was not selected for normality but contains mostly normal fetuses, as would be expected for a screening scenario: it does not contain enough serious abnormalities to make it possible to quantify the performance of our method in those cases. Where the credible interval for a biometric overlaps with a threshold for concern (often the 3rd and 97th percentiles) a manual measurement should be performed by the sonographer to confirm the finding and assist with any clinical decision-making.

Our system is designed to be robust to failures in individual frames, from plane misclassifications and failure in measurements in correctly classified frames. We do this both by acquiring large numbers of measurements and by explicitly modelling misclassifications within our expected measurement distribution (described in our ‘Methods’ section). In a practical real-time implementation of this method, a large number of frames must be processed every second. This means that additional post-processing steps, which may improve performance on some failure cases (such as those in Fig. 5) but add significant computational overhead, should be avoided in the design of such a system unless they can be optimised to the point that they are compatible with real-time operation.

We have tested our system using images from an unselected population of fetuses, almost all of which are normal. A potential future research direction would be to examine the performance of our methods on abnormal fetuses and establish their validity in that case. A prospective trial of this system to evaluate this is in progress.

In conclusion, this paper has demonstrated a novel method using machine learning to estimate fetal biometrics at the 20-week scan. The proposed method does not rely on measurements performed on individual manually-selected standard planes, as is the norm in both manual measurements and many commercial machine-assisted systems, but estimates biometrics across the entire scan. This avoids the biases exhibited by humans in plane selection and biometric measurement. We further present evidence that estimates produced using the proposed methods may be more consistent and repeatable than human measurements. This approach can also present other benefits that have not been quantified in this paper: by removing the need for sonographers to freeze the US stream and perform measurements, the system can improve sonographer focus and reduce the time needed to complete a FASP scan. A prospective trial of this system is in progress to establish whether this can realise those benefits and improve the detection of fetal abnormalities in a population selected for a higher incidence of abnormalities.

## Methods

### Datasets

The data for this work is taken from the iFIND project on fetal US (www.ifindproject.com), which recruited mothers attending a routine 20-week anomaly screening clinic in London, UK. Ten thousand volunteers gave consent to have their full 20-week US examinations (gestational age 18–22 weeks) recorded (King’s College London Research Ethics Committee reference 14/LO/1805). The examinations were conducted on identical US machines (GE Voluson E8) by 145 professional sonographers following FASP protocols between 2015 and 2020. The sonographer identified, labelled, and saved standard plane images and manually measured biometrics. To best reflect the screening population, all scans were included regardless of whether the scan was reported as normal or abnormal.

Although the same machines were used throughout this study, software updates during this period changed the video resolution and the interface presented by the machine. Recordings were split across three resolutions depending on the date on which they were acquired: 678 × 576, 980 × 784, and 1280 × 1024. These resolutions are evenly split across training, validation, and test folds.

Due to operator error, technical glitches, and patient withdrawal of consent, not all of these scans could be used in the dataset: 7309 video recordings of prenatal US scans were used for this study.

To maintain patient anonymity, personal information was removed from the scan recordings and each scan was labelled with a numerical iFIND ID, numbered sequentially. We created training/validation/test splits across the dataset-assigned subjects using the trailing digit of their iFIND ID. Scans with an iFIND ID with a trailing digit between 0 and 5 were used to train our models, 6 and 7 were used for validation, and 8 and 9 were held aside for testing and not used during training of any of our models. The demographic characteristics of patients across these folds, none of which showed statistically significant differences, are shown in Table 4.

**Table 4:** Patient characteristics and metadata across the data folds used for model development

Data fold	Train	Validation	Test	Lowest *p*-value
Total scans	4395	1457	1457	N/A
Abnormalities	528 (12.0%)	155 (10.6%)	163 (11.2%)	0.360
Live birth	4363 (99.2%)	1442 (98.9%)	1450 (99.5%)	0.375
Avg. GA	20 w 3 d	20w 3d	20w 3d	0.653
Avg. maternal age	32.3	32.6	32.5	0.193
Nonwhite	1352 (30.8%)	458 (31.4%)	418 (28.7%)	0.166
Avg. BMI (kg/m^2^)	24.7	24.9	24.9	0.448

None of the patient characteristics, including gestational age (GA), maternal body mass index (BMI), or percentage of abnormal scans were significantly different across the chosen folds.

In addition, some of our methods were also validated using a small number of paired scans from a smaller study^15^. Twenty-three volunteers were recruited after their 20-week anomaly scan for a follow-up scan. These ranged in gestational age from 20^+0^ to 24^+0^ gestational weeks (median 23GW), and had all screened as normal at their anomaly scan. These subjects gave their consent to have paired scans (encompassing the FASP planes and biometrics) conducted by separate sonographers on the same day, using a Philips EpiQ 7 US machine (King’s College London Research Ethics Committee reference 14/LO/1806). These scans were not used to train our models.

### Data labelling

**Fig. 6:**
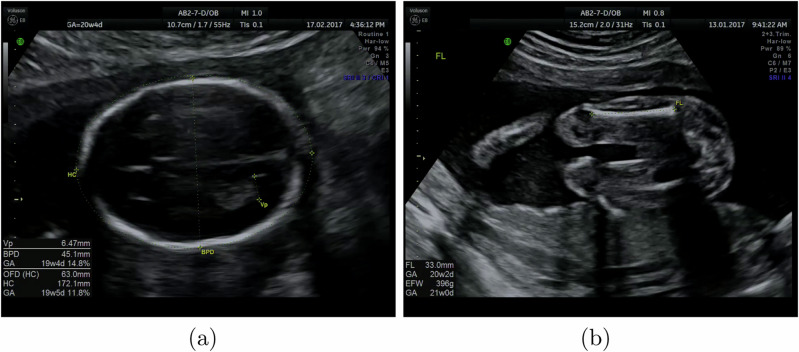
Example frames from routine ultrasound examinations with a sonographer’s annotations and calipers. Shown are **a** a Brain-TV image, with annotations for head circumference, biparietal diameter and posterior ventricle, **b** a femur image, with the femur length measured and annotated. These frames were acquired and saved by a sonographer, and we extracted their annotations to form part of our training set.

The iFIND dataset includes full recordings of examinations that were labelled during scanning with standard planes and biometrics according to the FASP standard as shown in Table 5 (note there is an additional background class for all other planes). These labels are present in the recording and were extracted automatically. Typically, a sonographer freezes the video stream once she identifies a suitable standard plane. Text labels and biometric measurements are then overlaid onto the frame, which is saved for the sonographer’s report. Example frames with these overlays are shown in Fig. 6.

**Table 5:** List of standard planes recommended under FASP

Standard plane	Description
*Head*	
Brain-CB	Transcerebellar view
Brain-TV	Transventricular view
*Heart*	
3VT	3-vessel + trachea
LVOT	left ventricular outflow tract
RVOT	left ventricular outflow tract
4CH	4-chamber view
*Other*	
Abdominal	
Femur	
Lips	
Kidneys	axial view
Profile	sagittal view
Spine-cor	coronal view
Spine-sag	sagittal view
*Background*	

We added a ‘Background’ label for frames in none of these views.

To find labelled planes in the recorded examinations, we automatically detected any pauses and freezes in the recordings, then used OCR software to extract any added text labels. For this project, we used the open-source Tesseract OCR package^19^ to read text labels. The text labels, including biometric labels (such as the Brain-TV view in Fig. 6a) were then associated with standard planes and manually checked for consistency. We consulted with sonographers to associate each OCR text label with a standard plane, ensuring that our dataset creation would be accurate. We manually checked a representative sample of 10% of the images obtained for each standard plane.

In the experiments performed in this paper, we removed segments where the sonographer froze the frame and added annotations. We did this by finding frames where over 95% of pixels did not change relative to the previous frame. This eliminates the sonographer’s own annotations from the recordings we used to test our methods.

We also labelled biometric measurements. In the UK, the FASP standard mandates a minimum of 5 biometric measurements per scan^2^ (additional measurements are required in some scans). These are measured across a range of standard planes, as shown in Table 6.

**Table 6:** Biometrics measured during the 20-week scan according to FASP guidelines, and the number of such labels present in the iFIND1 dataset

Biometric		Type	No. of labels
*Brain-TV*			
HC	Head circumference	Ellipse	8162
BPD	Biparietal diameter	Ellipse axis	8162
*Brain-CB*			
TCD	Transcerebellar diameter	Linear	6906
*Abdominal*			
AC	Abdominal circumference	Ellipse	6073
*Femur*			
FL	Femur length	Linear	5718

**Fig. 7:**
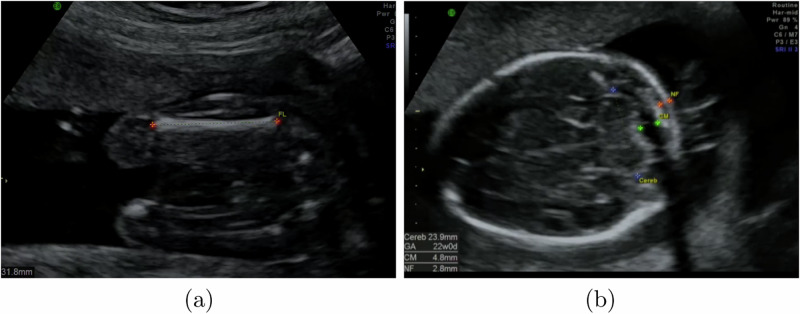
Example heatmap outputs of our CaliperNet annotation extraction CNN. Shown are extracted annotations for **a** a femur image, and **b** a brain-CB image with labels for the cerebellum, cisterna magna and nuchal fold. The cisterna magna and nuchal fold annotations were not used in this paper.

The calipers identifying a biometric are generally connected by a dotted line, with a text label next to them to identify the calipers (see Fig. 7). To extract these biometrics, we trained a simple U-Net based neural network which we called CaliperNet. We manually labelled 300 images of each biometric with the location of the calipers and trained a CNN to find calipers, with very good accuracy. We did not identify any failure cases in training: full training details and results can be found in Supplementary Material A.

### Biometric measurement

**Fig. 8:**
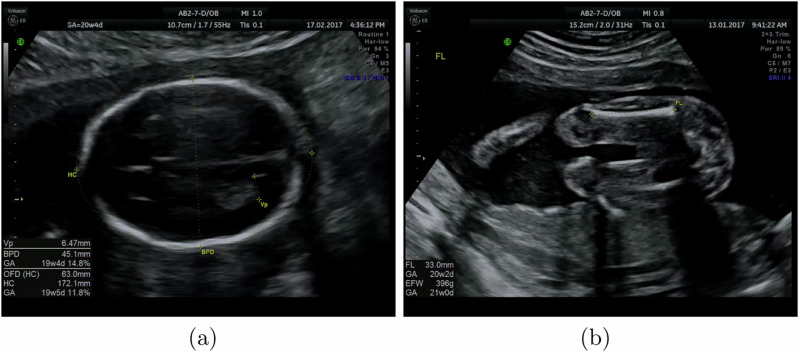
Examples of images and target annotations used in U-net segmentation training. Shown are **a** femur and **b** head views. The second row shows the training labels and the third row shows the CNN output overlaid on the image. For the head image, the HC output is shown in green and the BPD output is in pink.

To measure biometrics, we trained a U-Net segmentation network using binary cross-entropy loss^11^ to predict heatmaps constructed from the sonographer’s point annotations, extracted by CaliperNet, convolved with a Gaussian kernel. Figure 8 shows examples of training labels constructed this way. A separate CNN was trained for each standard plane, using the same U-Net architecture for each network and differing only in training planes and labels.

The U-Net architecture is a fully convolutional neural network based on an encoder-decoder architecture that is commonly used for segmentation in biomedical applications^20^. The architecture employed was the same as that proposed by Ronnerberger et al.^20^, using a learning rate of 10^−3^ and a batch size of 32 images. We employed data augmentation using random horizontal reflections, scaling up to ± 20%, rotations up to ±5°, and gamma correction, to simulate different probe positions and ultrasound machine gain settings. The networks we used were trained for 50 epochs on a single Nvidia GeForce RTX 3080 GPU, training in approximately 12 hours. All models were trained only on our training dataset, with no pretraining.

Biometrics were extracted from unseen images directly from the predicted heatmaps. For linear biometrics (FL and TCD), the Euclidean distance between the coordinates of the two highest maxima was used. For elliptical biometrics (HC and AC) we fit an ellipse to the heatmap using the least-squares criterion. The ellipse perimeter was then estimated using the formula recommended by the British Medical Ultrasound Society^21^.

Since BPD is mathematically equivalent to the minor axis of the ellipse used to segment the head, we calculated it directly from the head circumference label.

### Pixel size estimation

The method described above can find biometric endpoints on an image, but the measurements are given in pixels rather than millimetres. Moreover, pixel size can be changed during scanning by varying probe settings and machine zoom levels.

**Fig. 9:**
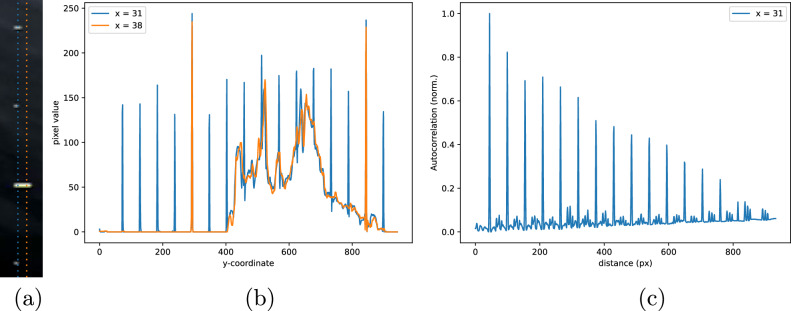
An illustration of the way we process the on-screen scale bar to extract the size of pixels in the image. **a** Part of a scale bar, cropped from an image frame. Two lines are overlaid upon it. **b** The pixel values along those two scan lines for the entire frame. **c** The autocorrelation of the pixel values of the blue line after it has been processed with a high-pass filter.

However, almost every frame shows a standard scale bar with prominent ticks at intervals *d*_bar_ = 50 mm, with smaller ticks every 10 mm and 5 mm^22^. These ticks are at predictable positions on the screen: they can be seen clearly by looking at pixel values *X*_*i*_ along the correct scan line (see Fig. 9). Ticks can sometimes blend into bright structures shown on the screen.

A simple, reliable and computationally efficient solution is to remove likely background signals from *X*_*i*_ by subtracting a 1D Gaussian filter, *G*_*x*_, with standard deviation *σ* empirically selected to be 3px and keeping only positive values:

2 $${X}_{i}^{{\prime} }=\max \left({X}_{i}-{X}_{i}* {G}_{x}(\sigma ),0\right),$$

We then computed the autocorrelation *R*_*XX*_ of the resulting filtered pixel values ${X}_{i}^{{\prime} }$ along the scan lines corresponding to the scale bar (see Fig. 9):

3 $${R}_{XX}(n)=\sum _{i}{X}_{i}^{{\prime} }{X}_{i+n}^{{\prime} }.$$

The first peak of the autocorrelation function corresponds to the spacing between axis ticks (in pixels). The size of individual pixels in millimetres *L*_*x*_ is given by

4 $${L}_{x}=\frac{{d}_{{\rm{bar}}}}{\max ({R}_{XX}(n))}.$$

To minimise quantisation noise, we estimate pixel size from the bars with the highest spacing.

Our external validation dataset was obtained with a different scanner model (Philips EpiQ 7), which had a slightly different interface with a different scalebar format. We used the same approach to measure pixel sizes, with slight adaptations for the different displayed ticks.

### Whole-scan biometric estimation

In clinical practice, only a few frames are labelled by the sonographer. Generally, no more than three measurements are taken of each biometric^2^ and the best subjective measurement is reported.

The automated methods described in the ‘Biometric measurements’ section can extract measurements in all identified frames in which a given biometric is visible, resulting in hundreds or thousands of measurements per biometric. These form sample populations from which we wish to infer a true value and estimate credible intervals.

However, for each biometric, there may be erroneous values caused by misclassification of the target anatomical planes or by failure modes of the biometric CNN. Any estimation needs to reject or at least minimise the effect of these out-of-distribution measurements to reliably converge on the true biometric value.

**Fig. 10:**
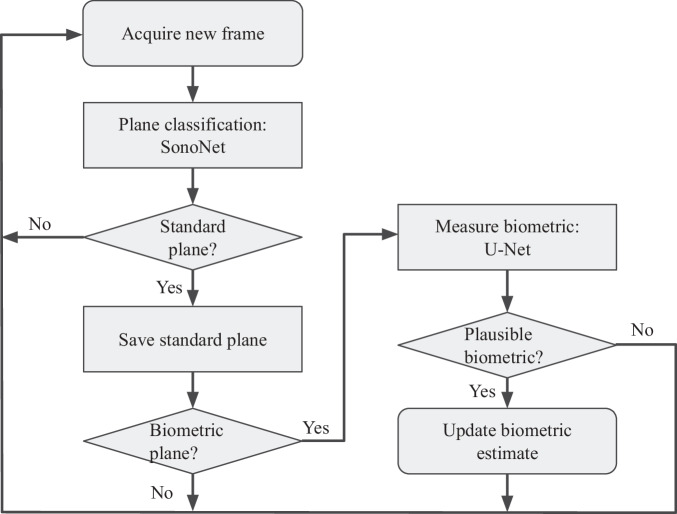
The pipeline which each frame in an US scan feed is processed by to estimate biometrics. Each new frame in a scan is classified by Sononet: most are not standard planes and require no further processing. Each frame that contains a standard plane is saved, and if it contains biometric measurements it is processed by U-Net to extract any visible biometrics. The overall biometric measurement is then updated.

Figure 10 shows our processing pipeline. Every frame from the US real-time image stream is processed by Sononet to identify and label standard planes. Following Baumgartner et al.^7^, each frame is resized to a resolution of 288 × 224 and converted to greyscale.

Any frame identified with >95% confidence as a standard plane with a FASP biometric is then processed by our CNNs to obtain a biometric measurement. The native frame is subsampled to a 384 × 288 resolution, with interface elements removed, and converted to greyscale and processed as described above.

A quick check is then applied: if the proposed measurement is anatomically implausible (lower than the 3rd centile of the biometric at GA −3 weeks or higher than the 97th centile at GA +3 weeks), it is rejected. Furthermore, where the CNN’s output does not allow an ellipse to be fitted or endpoints to be returned (for instance, where one distinct maximum is found, making it impossible to find two endpoints) the output is discarded.

These two filters (of plane-classification confidence and biometric measurements) may be undesirable for single-frame comparisons, as they discard some data. However, when estimating biometrics across a whole scan several hundred measurements of each biometric are often recorded: if some fraction of these are discarded, this only has a small impact on data availability. Supplementary Material B details the proportion of biometric measurements discarded.

Repeated measurements of the same biometric in different frames should cluster around a mean value. We modelled this using a normal distribution ${\mathcal{N}}\left(\mu ,{\sigma }^{2}\right)$, with mean *μ* (if unbiased, this should be the true biometric value) and variance *σ*^2^(this depends on the precision of the biometric measurements).

Misclassified planes also return a biometric measurement and the biometric measurement process itself may fail even in a correctly identified plane. Since we have no information with which to model these failures, we treat them as random and use a uniform distribution $U\left(a,b\right)$, over the full biological limits *a* and *b*, outside of which a measurement is rejected.

**Fig. 11:**
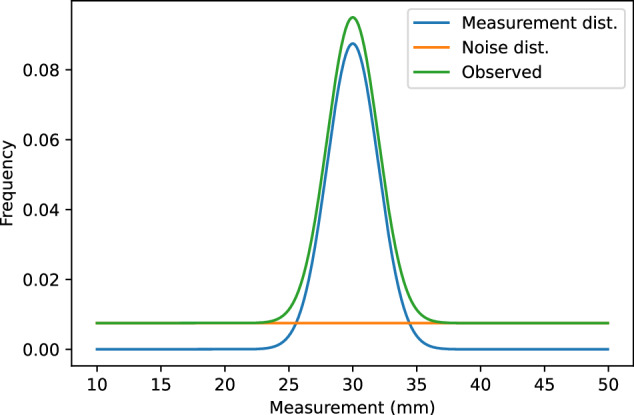
An idealised distribution of biometrics for a biometric with true value 30mm. The observed distribution of measurements is a sum of a measurement distribution (modelled as a Gaussian distribution) and a nuisance distribution of outliers (modelled as a uniform distribution).

Thus, the observed distribution *D*_*o*_ of measurements will be a weighted sum of the measurement distribution ${\mathcal{N}}\left(\mu ,{\sigma }^{2}\right)$ (for correct classifications) and a nuisance distribution $U\left(a,b\right)$ (for misclassifications). This can be represented by

5 $${D}_{o}={P}_{t}{\mathcal{N}}\left(\mu ,{\sigma }^{2}\right)+(1-{P}_{t})U\left(a,b\right),$$

where *P*_*t*_ represents the proportion of valid measurements. Figure 11 shows how we model the measurement distribution.

This equation contains several unknowns *P*_*t*_, *μ* and *σ*^2^, which can be estimated iteratively using a Bayesian approach, but each must be initialised.

To perform a Bayesian estimation, *P*_*t*_ needs to be initialised to a prior probability (in our approach, we set ${P}_{{t}_{0}}=0.75$ for all biometrics, but it should converge on the true probability from any starting point other than 0 or 1), along with a prior weight *W*_0_. Prior estimates of the distribution parameters *μ*_0_ and ${\sigma }_{0}^{2}$ need to be set, along with appropriate prior weights *W*_*μ*,0_ and ${W}_{{\sigma }^{2},0}$. The choice of parameter *W*_0_ influences the speed of convergence: the algorithm will converge on the true value of *P*_*t*_ regardless of what *W*_0_ is set to, but setting it too high can cause it to converge slowly while setting it too low can cause the estimate of *P*_*t*_ to oscillate inappropriately.

For each new measurement *x*_*i*_, we estimate the likelihood of it being a true measurement *T* or a nuisance sample $\overline{T}$ using Bayes’ rule:

6 $$P(T| {x}_{i})=\frac{P({x}_{i}| T){P}_{t}}{P({x}_{i}| \overline{T})(1-{P}_{t})}$$

where *P*(*x*_*i*_∣*T*) is the value of ${\mathcal{N}}\left(\mu ,{\sigma }^{2}\right)$ at *x*_*i*_, and $P({x}_{i}| \overline{T})$ is the value of $U\left(a,b\right)$ at value *x*_*i*_. This returns *P*(*T*∣*x*_*i*_), the probability that this measurement was sampled from the true measurement distribution.

Finally, *P*_*t*_ and the distribution parameters *μ*, *σ*^2^ can be updated for this biometric using a weighted cumulative average:

7 $${P}_{{t}_{i}}=\frac{P(T| {x}_{i})+{W}_{i-1}{P}_{{t}_{i-1}}}{{W}_{i-1}+1}$$

8 $${\mu }_{i}=\frac{{x}_{i}P(T| {x}_{i})+{W}_{\mu ,i-1}{\mu }_{i-1}}{P(T| {x}_{i})+{W}_{\mu ,i-1}}$$

9 $${\sigma }_{i}^{2}=\frac{{({x}_{i}-{\mu }_{i-1})}^{2}P(T| {x}_{i})+{W}_{{\sigma }^{2},i-1}{\sigma }_{i-1}^{2}}{P(T| {x}_{i})+{W}_{{\sigma }^{2},i-1}}$$

and their weighting factors can be updated using

10 $${W}_{i}={W}_{i-1}+1$$

11 $${W}_{\mu ,i}={W}_{\mu ,i-1}+P(T| {x}_{i})$$

12 $${W}_{{\sigma }^{2},i}={W}_{{\sigma }^{2},i-1}+P(T| {x}_{i}).$$

As the noise distribution is taken to be uniform, there is no need to update estimates of its terms.

The estimates for *μ* and *σ*^2^ are updated in real-time and independently. At any given moment, the estimate of *μ* is the best estimate of the true value of the biometric of interest. Meanwhile, the estimate of *σ*^2^ can be used to calculate the standard error, ${\hat{\sigma }}_{i}$, of that estimate to find credible intervals for the biometric, given by

13 $${\hat{\sigma }}_{i}=\frac{{\sigma }_{i}}{\sqrt{{W}_{{\sigma }^{2},i}}}.$$

The 95% credible interval is given by the range ${\mu }_{i}\pm 2{\hat{\sigma }}_{i}$.

## Supplementary information

Supplementary material

## Data Availability

The US scan recordings used to train the CNNs used in this paper were collected with an ethical requirement for patient data to remain confidential. As such, the scan data cannot be made publicly available. The raw and processed CNN outputs used to construct our results are available from the corresponding author on reasonable request.

## Code availability

The code used for this paper to process our CNN’s outputs and generate overall estimates from video data is available at https://gitlab.com/sonographai/biometric-estimation-code-release.
