# Supplementary material for: Whole examination AI estimation of fetal biometrics from 20-week ultrasound scans
Source: NPJ Digit Med. 2025 Jan 11;8:22. doi: 10.1038/s41746-024-01406-z (PMC11724865; doi:10.1038/s41746-024-01406-z)
Supplement: Supplementary file 2 — Supplementary material [file 41746_2024_1406_MOESM1_ESM.pdf]

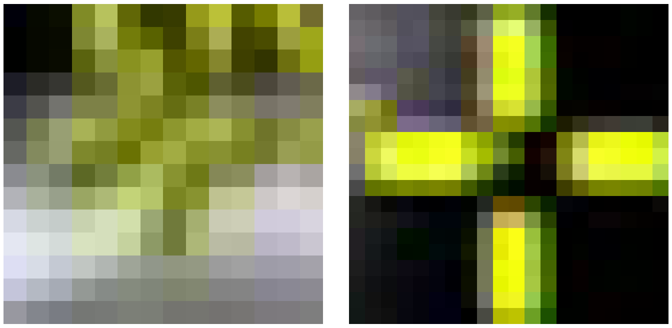

Supplementary Figure 1: Zoomed examples of two calipers in the training set. These are at different scales, with different backgrounds and levels of overlap with other labels: it is difficult to design a machine method that can extract both reliably.

Supplementary Information A. CNN for caliper localisation

Supplementary Information A.1. Introduction

For this project, we used the sonographer's annotations taken during their ultrasound scans as a source of data labels. These are readily visible in the scan and trivial for a human to detect: the calipers places by sonographers are prominent in their shape and colour. However, there were too many calipers to feasibly extract manually: approximately 40,000 labelled images were used in this paper.

Similarly, it is difficult to use a machine algorithm to extract these calipers automatically. Though all ultrasound scans were performed on identical hardware, there were several software updates over the course of the iFIND study, which changed the resolution and rendering of calipers. These weren't always consistently rendered on the machine, as they appeared to be interpolated and therefore displayed differently. Furthermore, calipers often overlap with text annotations or other calipers, making it difficult to reliably distinguish them. Figure 1 shows two very different caliper shapes on different backgrounds.

We used a simple ML tool, which we called CaliperNet, to extract these labels automatically.

Supplementary Information A.2. Labels and training

We used a sample of 300 images per standard plane to be manually annotated with caliper locations. We obtained these labels from a single human annotator on Labelbox [1]. Caliper locations are exact, so we expect very little labelling noise.

Labelbox does not have a native ellipse annotation facility, so to trace ellipse labels we asked the annotator to pinpoint five points along the perimeter of the ellipse: five points are sufficient to fully constrain an ellipse's parameters. Training labels were generated in the same way as in the main paper: for length biometrics, the endpoints were convolved with a Gaussian kernel to generate a heatmap. For ellipse biometrics, the outline of the ellipse was convolved with a Gaussian kernel.

We used the same U-Net architecture as for our full biometric models to train CaliperNet. The only difference was the retention of image colour channels: calipers in the GE interface are coloured yellow, while the rest of an ultrasound image is usually in greyscale (though the operator can change the colour map), so important information is carried by the colour channel.

We trained three separate models: one to extract ellipse annotations (such as HC and AC), one to extract caliper locations in the femur image, and one to extract TCD locations in transcerebellar brain images. The TCD model had to be trained separately as for most images in that plane, the sonographer took three annotations and

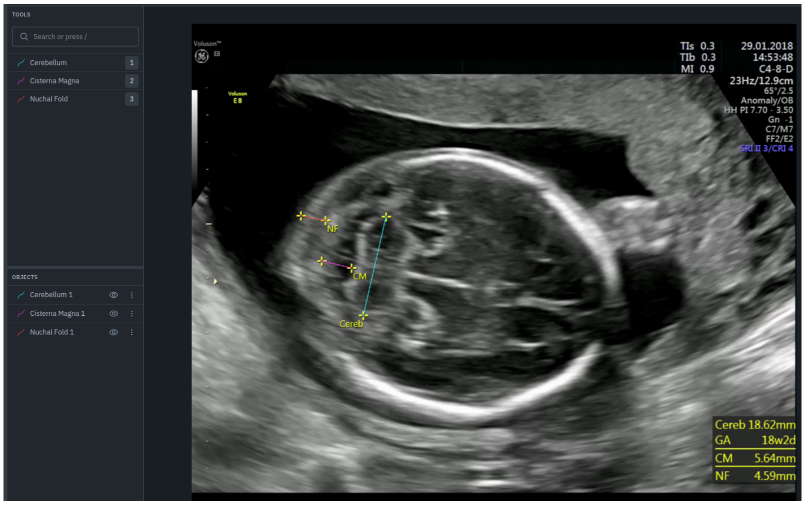

Supplementary Figure 2: The interface of Labelbox which we used to annotate calipers. This screenshot includes with an example annotation of a transcerebellar image with three manual annotations.

placed three sets of calipers: the TCD, the cisterna magna (CM), and the nuchal fold (NF)[1]. Figure 2 shows an example image with three sets of calipers. For TCD calipers, we generated multi-channel labels, with one channel per annotated structure. Not all images in this standard plane had all three biometrics labelled: if calipers were missing, the relevant channel was left blank.

Training was performed using a 75:10:15 train:validation:test split for images. We used the same hardware to train CaliperNet models as the models described in the main paper.

Supplementary Information A.3. Results

Biometric	Bias (%)	MSE (%)	Dice coefficient
HC	-0.01%	0.30%	0.821
AC	0.00%	0.34%	0.819
FL	+0.09%	0.66%	0.861
TCD	+0.07%	0.95%	0.946

Supplementary Table 1: Results for each CaliperNet trained on one biometric.

Table 1 shows the performance of CaliperNet on our test dataset across the structures of interest. There is very little error, showing that this is a fairly straightforward task for these networks. Often, the pixel localisations are exactly the same: the errors are usually on the order of ±1px in specific pixel locations. The 'Dice coefficient'

[1] Of these, the TCD measurement is in the FASP standard and is required to always be measured. Nuchal fold must be measured only if it is abnormally thick, while cisterna magna was in a previous version of the FASP standard but is no longer required.

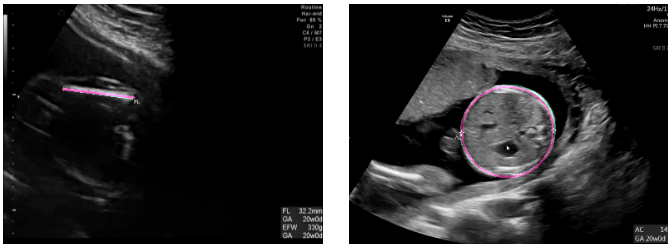

(a) (b)

Supplementary Figure 3: The annotations with the largest % errors in the test sets. Green is the ground-truth annotation and pink are the CaliperNet outputs. Shown in (a) femur (length error of about 1%), and (b) abdomen (about 2%). This shows that CaliperNet is very accurate with this task.

column measures how well the network output maps replicate the training labels. While it is high, it is not perfect: this may be because the limited training data makes it difficult for the CNN to learn to replicate the Gaussian kernels consistently. Nevertheless, the regressed endpoints and ellipses remain reliable.

There were no failure cases across our test dataset. Figure 3 shows the images with the largest errors (-1% and +2%) in biometric between the manual annotation of the calipers and the CaliperNet output. In both cases, the difference was of 1px in endpoint localisation - within labelling noise. This reflects the fact that caliper localisation is a trivial task for humans, and is not a challenging task for CNNs. Therefore, we can have confidence in the labels generated by CaliperNet and can use them to generate training labels for our biometric CNNs.

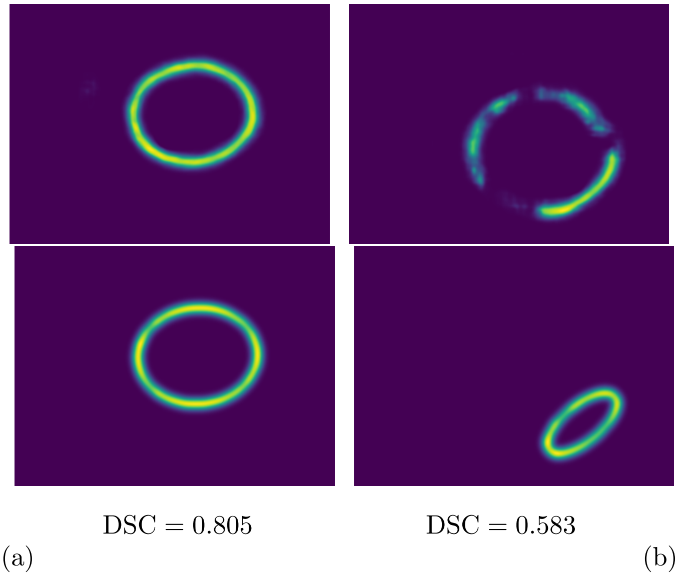

Supplementary Figure 4: Two comparisons of the output heatmap from a CNN and the resulting 'reconstructed' ellipse. (a) a success case with DSC = 0.805, (b) a failure case with DSC = 0.583.

Supplementary Information B. Additional performance metrics for biometric CNNs

Supplementary Information B.1. Introduction

Four additional metrics were used to measure the performance of our biometric CNNs and to filter for failure cases at test time. These could give substantial indicators of failure cases, making them useful to reject failure cases and reduce noise in our whole-video estimates. One metric, the Dice similarity coefficient of a reconstructed heatmap, was used to measure the quality of fit of derived ellipses and points from the CNN's heatmap: where this was too low, the fit was deemed of insufficient quality to accept the resulting measurement. The other was the eccentricity of the output ellipses for head and abdominal circumference measurements: where this was anatomically implausible, the measurements were rejected. We also examined the loss curves for our biometric CNNs during training and conducted a subgroup analysis to check for any difference in performance between different ethnic subgroups.

Supplementary Information B.2. Dice similarity coefficient

The Dice similarity coefficient (DSC) is a measure of overlap between two sets defined as

$$\mathrm{DSC}(X, Y) = \frac{2\,|X \cap Y|}{|X| + |Y|} \tag{B.1}$$

and is often used in image segmentation tasks to measure how closely a segmentation follows the ground truth.

Our biometric CNNs output heatmaps based on endpoints and ellipses, as described in the Methods section of the main paper. These are then processed to return a biometric measurement: for linear measurements, endpoints are found from local maxima of the heatmap; for ellipses, an ellipse is fit using least-squares to the heatmap.

This can then be checked by generating a heatmap from the resulting annotation, using the same process described in the main paper. The two heatmaps should match exactly in the case of a perfect output. In reality,

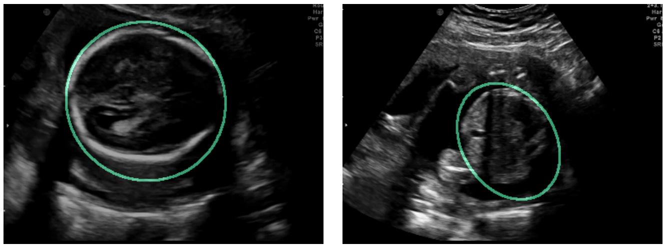

$e = 0.242$ (a) $e = 0.670$ (b)

Supplementary Figure 5: Two examples of biometric failure cases which violate the eccentricity constraint and are therefore not included in the overall estimate for a scan. These examples are in (a) the head (eccentricity too low), and (b) the abdomen (eccentricity too high).

there is always some deviation, but when the reconstructed ellipse faithfully follows the output heatmap, the resulting DSC is high.

We found that a DSC < 0.6 between the output heatmap and the reconstruction indicated a poor reconstruction of the CNN output. Therefore, we discarded any proposed reconstructions that fell below that threshold, as the output would be very noisy and inaccurate in following anatomical boundaries.

Figure 4 shows two example cases of reconstructed ellipses based on an output heatmap. (a) shows a success case, where the reconstructed ellipse closely follows the output heatmap: this would be accepted. (b) shows a case where the output heatmap is quite noisy and results in poor fit of the reconstructed ellipse. This has low DSC and therefore is rejected.

Supplementary Information B.3. Ellipse eccentricity

For ellipse biometrics (HC and AC), another relevant metric to consider at test time is eccentricity.

$$e = \sqrt{1 - \frac{a^2}{b^2}} \tag{B.2}$$

where a is the minor axis of the ellipse and b is the major axis. For the head, a is the BPD measure and b is the OFD (occipito-frontal diameter). While this is a commonly used mathematical measure, in obstetrics the more common measure for head measurements is the **cephalic index**, defined as

$$CI = \frac{a}{b} \times 100. \tag{B.3}$$

In the case of an ellipse, there is a 1:1 correspondence between the two measures, where

$$e = \sqrt{1 - \left(\frac{CI}{100}\right)^2}. \tag{B.4}$$

In the 17-22 week range of gestational age, the mean cephalic index has been reported as 75.9, with a standard deviation of 3.7 [2]. Therefore, an anatomically plausible range of cephalic indices is $65 - 90$, which corresponds to an eccentricity range of $0.43 - 0.76$. Any ellipse with an eccentricity outside of this range is likely to be a failure mode of the biometric CNN.

In the final product using this CNN, we relaxed this constraint further to an eccentricity range of $0.25 - 0.8$. This was to restrict rejections to clear failures, allowing a large margin for biological variation.

The fetal abdomen has somewhat different morphology. It is typically round, with a very low eccentricity. We constrained it to have $e < 0.6$ for the purposes of our software - any abdominal ellipse with a higher eccentricity was considered biologically implausible and rejected.

Figure 5 shows two examples of drawn ellipses which violate this eccentricity constraint. Both are clear failures, where the ellipse does not follow anatomical boundaries. The addition of our constraint rejects the measurements from these ellipses as noise, even if the circumferences appear biologically plausible.

Supplementary Information B.4. Plane classification confidence

Standard plane	Frames >95% confidence (%)
Brain-TV	83.5%
Brain-CB	82.1%
Abdominal	73.2%
Femur	66.4%

Supplementary Table 2: Proportion of frames in the four biometric standard planes which are classified with >95% softmax confidence.

The main paper describes one filtering method: only frames identified with $> 95\%$ confidence as a standard plane with a FASP biometric are processed by biometric networks. This somewhat reduces the number of frames passed to biometric networks and therefore the number of samples of each biometric.

Table 2 shows the proportion of frames classified as belonging to each of the four biometric standard planes with sufficient confidence for a measurement. In all cases, only a minority of frames are discarded.

Supplementary Information B.5. Training loss curves

It is important to examine training and validation loss curves while training a CNN to ensure that it is converging and not overfitting.

Figure 6 shows training and validation loss curves for our femur model. The validation loss declines smoothly until it converges on a steady value, which is a sign of convergence of training. The validation loss is also consistently lower than the training loss, which is also reassuring: the training set is augmented using methods described in the main paper, which makes predictions on it more challenging than on the unaugmented validation set. It is important to note that training loss is calculated for each batch, so this introduces some noise in the measure. Overall this training curve appears well-behaved and reassuring. Training curves for the other biometric models show a similar pattern.

Supplementary Information B.6. Performance on demographic subgroups

Table 3 shows the difference in our biometric models' performance across ethnic groups in the videos in our test set.

None of the differences between demographic groups are statistically significant with $p < 0.05$, except for the difference in femur length error between white and Asian subjects, which is significant with $p = 0.03$. There is a large number of possible comparisons across different biometrics and demographics, this difference is not present in other biometrics (even directionally), and there is no prior reason to expect this difference. We therefore think it is likely that this is a random statistical fluctuation and that there are no real differences in biometric performance across demographic subgroups.

Head circumference			
Ethnicity	Number	Bias (mm)	MSD (mm)
All	609	-0.32 (-0.18%)	3.23 (1.84%)
White	456	-0.30 (-0.18%)	3.23 (1.83%)
Black	70	-0.42 (-0.23%)	3.70 (2.11%)
Asian	57	-0.53 (-0.30%)	2.88 (1.66%)
Other	26	+0.12 (+0.07%)	2.73 (1.59%)

Abdominal circumference			
Ethnicity	Number	Bias (mm)	MSD (mm)
All	962	-0.50 (-0.32%)	5.55 (3.59%)
White	710	-0.53 (-0.34%)	5.50 (3.55%)
Black	117	-0.63 (-0.40%)	5.97 (3.96%)
Asian	95	-0.86 (-0.54%)	4.63 (3.03%)
Other	40	+1.22 (+0.77%)	6.97 (4.38%)

Femur length			
Ethnicity	Number	Bias (mm)	MSD (mm)
All	858	-0.51 (-1.58%)	1.63 (4.99%)
White	632	-0.53 (-1.65%)	1.61 (4.94%)
Black	112	-0.72 (-2.12%)	1.81 (5.51%)
Asian	82	-0.16 (-0.48%)	1.60 (4.83%)
Other	32	-0.32 (-1.07%)	1.41 (4.34%)

Transcerebellar diameter			
Ethnicity	Number	Bias (mm)	MSD (mm)
All	492	+0.27 (+1.31%)	0.91 (4.38%)
White	358	+0.27 (+1.33%)	0.87 (4.20%)
Black	57	+0.08 (+0.42%)	1.02 (4.83%)
Asian	53	+0.45 (+2.19%)	1.06 (5.08%)
Other	24	+0.21 (+1.10%)	0.84 (4.12%)

Supplementary Table 3: Performance of our biometric models on different ethnic subgroups in the test set.

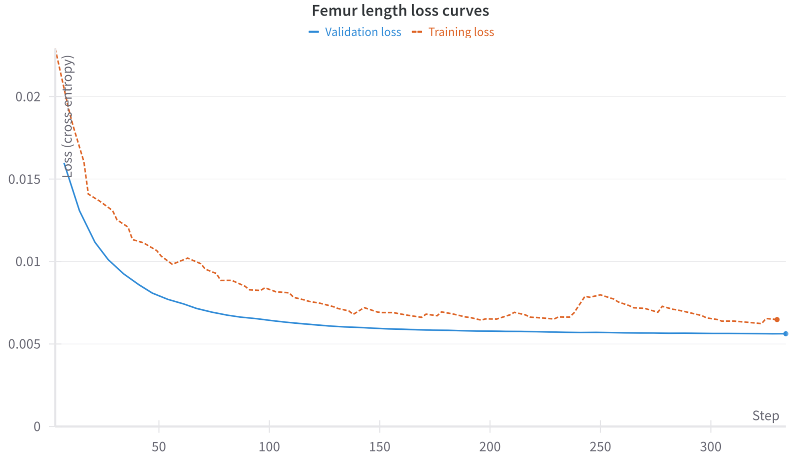

Supplementary Figure 6: Training and validation loss curves for the 'femur length' model. The training loss was calculated per-batch, which introduces greater noise in the measured loss.

Measure	Output (mm)
Bias (%)	+0.53mm (0.3%)
MSE (mm)	2.40mm (1.4%)
MAE (mm)	1.79mm (1.0%)

Supplementary Table 4: Performance of our HC model on the HC18 public challenge dataset.

Supplementary Information C. External validation on public dataset

Supplementary Information C.1. Data description

The networks described in this paper were trained on ultrasound scans recorded at a single site on one device. Therefore, the validity of the network's predictions outside of this domain needs to be investigated. The main paper describes how we validated our methods on scans acquired with a different ultrasound machine at the same site. However, this is a very small number of scans.

We therefore validated these outputs on a publicly available dataset, the HC18 dataset of fetal head circumference measurements [3]. This is a public dataset that was used for a MICCAI challenge in 2018, acquired in the Netherlands. Its 'training' set consisted of 999 2D images of the fetal head, along with annotations of the head circumference measurement as well as the measured HC in millimetres. To provide an accurate picture of the robustness of our methods to domain shift, we did not train our models on any data from this set: we only used these images to test the models that we trained on our data.

Supplementary Information C.2. Dataset filtering and processing

The dataset spans a gestational age range of 11-37 gestational weeks, with most of the images collected around 12GW and 20GW to correspond with regular screening scans. We restricted the data we tested our methods on to

HC range	50-100mm	100-150mm	150-200mm	200-250mm	250-300mm	300-350mm
Count	104	55	536	66	98	49
Bias (%)	+3.62mm (4.6%)	+1.58mm (1.4%)	+0.53mm (0.3%)	+2.42mm (1.1%)	+2.44mm (0.9%)	+2.85mm (0.9%)
MSE (mm)	15.85mm (21.1%)	4.77mm (4.1%)	2.40mm (1.4%)	4.72mm (2.0%)	11.89mm (4.1%)	6.60mm (2.1%)
MAE (mm)	7.20mm (9.4%)	3.03mm (2.6%)	1.79mm (1.0%)	3.49mm (1.5%)	5.40mm (1.9%)	4.90mm (1.5%)

Supplementary Table 5: Performance of our HC model on different parts of the HC18 challenge dataset, including data outside of our training distribution.

images with an HC between 150-200mm ($n = 547$ volumes), roughly corresponding to the expected range in the gestational age range in our training dataset.

We applied the same preprocessing to these images at the input to our network as we did for our own dataset: we resized the images to $384 \times 288 \times 1$.

Supplementary Information C.3. Results within training distribution

For 2 images in the dataset (0.4%), the biometric network did not return a valid ellipse: no measurement was returned for these images. This would not be a valid output for the original challenge for which this dataset was designed, as an HC measurement is expected for every image. However, this is a desired outcome for the use for which our models were designed: in an ultrasound video stream, a very large number of frames of each biometric are available. If the measurement method fails on a small minority of the frames, it is better to return no measurement than to return a potentially flawed measurement that may affect the final estimate.

For the remaining 545 images (99.6%), an HC measurement could be obtained. The results for that set are shown in Table 4, and are broadly similar to those obtained on our own dataset. There does not appear to be any significant domain shift from

Any comparisons with published results for this dataset must be interpreted with caution. We did not train our models on their supplied training data, but on our own dataset. We also excluded a large subset of the dataset whose gestational age did not align with that of our training set: a direct comparison with public leaderboard results can therefore only be qualitative. Nonetheless, on the metric used to construct the public leaderboard (MAE in HC measurement) our measure of 1.79mm would rank in the 98th percentile of challenge submissions.

This result shows that our models achieve good performance in public datasets that were not collected by our group.

Supplementary Information C.4. Results outside of the training distribution

We also measured the performance of our model on other parts of the HC18 dataset, which covered ranges of head circumference corresponding to gestational ages outside of our training dataset. The results of our model are shown in Table 5.

As expected, the best results are found in the HC range corresponding to our training distribution (and already discussed above). Gestational ages very different from the training distribution (with correspondingly different HC values) lead to a significant degradation in performance, which increases with the increase in difference from the training range. It's worth noting that the MSE degrades more than the MAE value for large differences in gestational age, which suggests there is a significant increase in catastrophic model failures in those age ranges.

This emphasizes that this model should only be used for gestational ages within its training distribution, where its outputs are most reliable.

Supplementary References

[1] "Labelbox | data-centric AI platform for building intelligent applications."

[2] S. Constantine, A. Kiermeier, and P. Anderson, "The normal fetal cephalic index in the second and third trimesters of pregnancy," *Ultrasound Quarterly*, vol. 36, pp. 255–262, 2020.

[3] T. L. A. van den Heuvel, D. de Bruijn, C. L. de Korte, and B. van Ginneken, "Automated measurement of fetal head circumference using 2D ultrasound images," *PloS one*, vol. 13, p. e0200412, 2018.
